# Supplementary material for: Assessment of environmental risk factors for blastomycosis during a large outbreak at a Michigan paper mill
Source: PLoS One. 2025 Sep 23;20(9):e0332398. doi: 10.1371/journal.pone.0332398 (PMC12456783; doi:10.1371/journal.pone.0332398)
Supplement: S1 Fig — DAG, made in DAGitty v3.1, of proposed causal relationships between work locations and environmental conditions at the mill and blastomycosis. Blastomyces exposure was unobserved. For associations between work location and blastomycosis, the minimum sufficient adjustment variables were sex and mill tenure. For associations between environmental conditions and blastomycosis, the minimum sufficient adjustment variables were sex, mill tenure, and work location. (PDF) [file pone.0332398.s001.pdf]

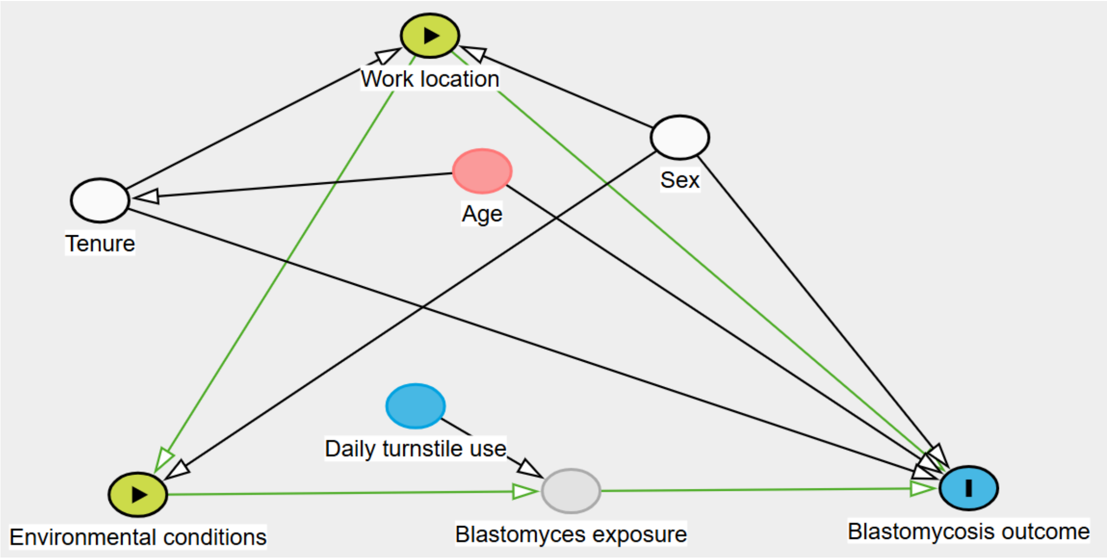

Supplemental Figure 1. Directed acyclic graph (DAG). DAG made in DAGitty v3.1, of proposed causal relationships between work locations and environmental conditions at the mill and blastomycosis. *Blastomyces* exposure was unobserved. For associations between work location and blastomycosis, the minimum sufficient adjustment variables were sex and tenure. For associations between environmental conditions and blastomycosis, the minimum sufficient adjustment variables were sex, tenure, and work location.

DAG model code:

```
dag {
bb="0,0,1,1"
"Blastomyces exposure" [latent,pos="0.512,0.672"]
"Blastomycosis outcome" [outcome,pos="0.870,0.668"]
"Daily turnstile use" [pos="0.372,0.556"]
"Environmental conditions" [exposure,pos="0.122,0.677"]
"Work location" [exposure,pos="0.385,0.050"]
Age [pos="0.432,0.235"]
Sex [adjusted,pos="0.610,0.189"]
Tenure [adjusted,pos="0.088,0.275"]
"Blastomyces exposure" -> "Blastomycosis outcome"
"Daily turnstile use" -> "Blastomyces exposure"
"Environmental conditions" -> "Blastomyces exposure"
"Work location" -> "Blastomycosis outcome"
"Work location" -> "Environmental conditions"
Age -> "Blastomycosis outcome"
Age -> Tenure
Sex -> "Blastomycosis outcome"
Sex -> "Environmental conditions"
Sex -> "Work location"
Tenure -> "Blastomycosis outcome"
Tenure -> "Work location"
}
```
