# Supplementary material for: Assessment of environmental risk factors for blastomycosis during a large outbreak at a Michigan paper mill
Source: PLoS One. 2025 Sep 23;20(9):e0332398. doi: 10.1371/journal.pone.0332398 (PMC12456783; doi:10.1371/journal.pone.0332398)
Supplement: S2 Fig — Primary work location at the mill was reported by workers from October 2022–April 2023. (PDF) [file pone.0332398.s002.pdf]

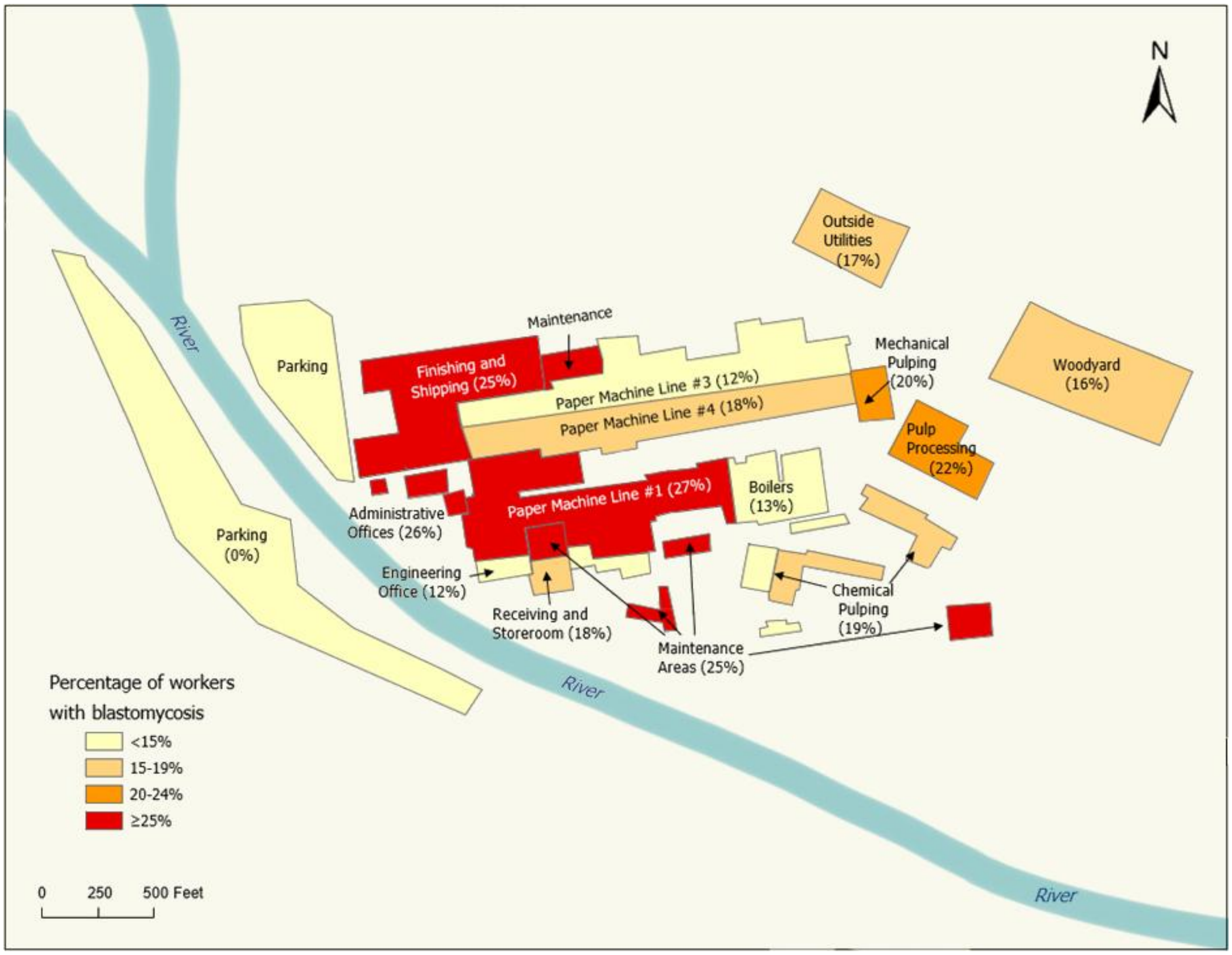

Supplemental Figure 2. Prevalence of blastomycosis based on the primary work location. Primary work location at the mill was reported by workers from October 2022–April 2023.
