# Supplementary material for: Assessment of environmental risk factors for blastomycosis during a large outbreak at a Michigan paper mill
Source: PLoS One. 2025 Sep 23;20(9):e0332398. doi: 10.1371/journal.pone.0332398 (PMC12456783; doi:10.1371/journal.pone.0332398)
Supplement: S1 Table — (PDF) [file pone.0332398.s003.pdf]

Supplemental Table 1. Associations between primary work location at the mill and blastomycosis.

Primary work location[a]	Unadjusted model		Adjusted model[b]	
	Prevalence ratios (PR) of blastomycosis	95% confidence intervals (CI)	PR of blastomycosis	95% CI
Administrative offices	1.38	(0.87, 2.20)	1.47	(0.91, 2.37)
Boilers	0.70	(0.33, 1.45)	0.69	(0.33, 1.41)
Paper Machine Line #1	1.46	(0.99, 2.16)	1.47	(0.99, 2.17)
Paper Machine Line #3	0.66	(0.37, 1.19)	0.65	(0.36, 1.18)
Paper Machine Line #4	0.98	(0.62, 1.55)	0.99	(0.63, 1.57)
Engineering office	0.62	(0.22, 1.70)	0.48	(0.14, 1.67)
Maintenance areas	1.34	(0.79, 2.27)	1.39	(0.81, 2.38)
Outside utilities	0.89	(0.27, 2.94)	0.88	(0.27, 2.85)
Finishing and shipping	1.36	(0.85, 2.20)	1.39	(0.86, 2.27)
Pulp processing	1.19	(0.38, 3.77)	1.25	(0.40, 3.92)
Chemical pulping	1.03	(0.48, 2.21)	1.02	(0.47, 2.20)
Mechanical pulping	1.07	(0.33, 3.45)	1.04	(0.33, 3.32)
Receiving and storeroom	0.97	(0.30, 3.18)	1.15	(0.37, 3.60)
Woodyard	0.85	(0.44, 1.66)	0.85	(0.44, 1.64)

[a] Reference is the overall prevalence, i.e., grand mean, of blastomycosis for all areas of the mill.
[b] Adjusted for tenure and sex.
