# Supplementary material for: Assessment of environmental risk factors for blastomycosis during a large outbreak at a Michigan paper mill
Source: PLoS One. 2025 Sep 23;20(9):e0332398. doi: 10.1371/journal.pone.0332398 (PMC12456783; doi:10.1371/journal.pone.0332398)
Supplement: S3 Fig — Blastomycosis illness onset dates were plotted by the primary work location of workers’ current or most recent job. Illness onset dates were available for 112 mill workers who participated in the NIOSH medical survey. The average blastomycosis illness onset date was March 4, 2023. Counts less than five were not reported to avoid identification of workers. (PDF) [file pone.0332398.s004.pdf]

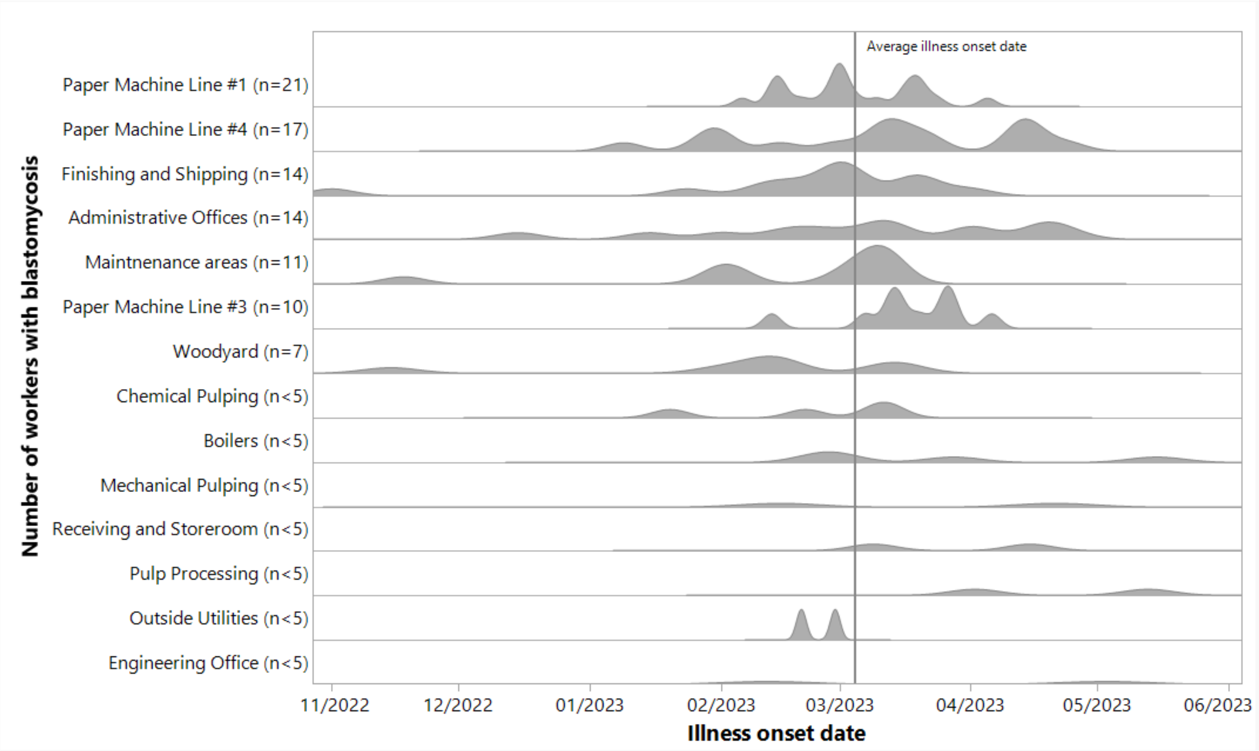

Supplemental Figure 3. Number of workers with blastomycosis, by illness onset date. Blastomycosis illness onset dates were plotted by the primary work location of workers' current or most recent job. Illness onset dates were available for 112 mill workers who participated in the NIOSH medical survey. The average blastomycosis illness onset date was March 4, 2023. Counts less than five were not reported to avoid identification of workers.
