# Supplementary material for: Assessment of environmental risk factors for blastomycosis during a large outbreak at a Michigan paper mill
Source: PLoS One. 2025 Sep 23;20(9):e0332398. doi: 10.1371/journal.pone.0332398 (PMC12456783; doi:10.1371/journal.pone.0332398)
Supplement: S4 Fig — (PDF) [file pone.0332398.s005.pdf]

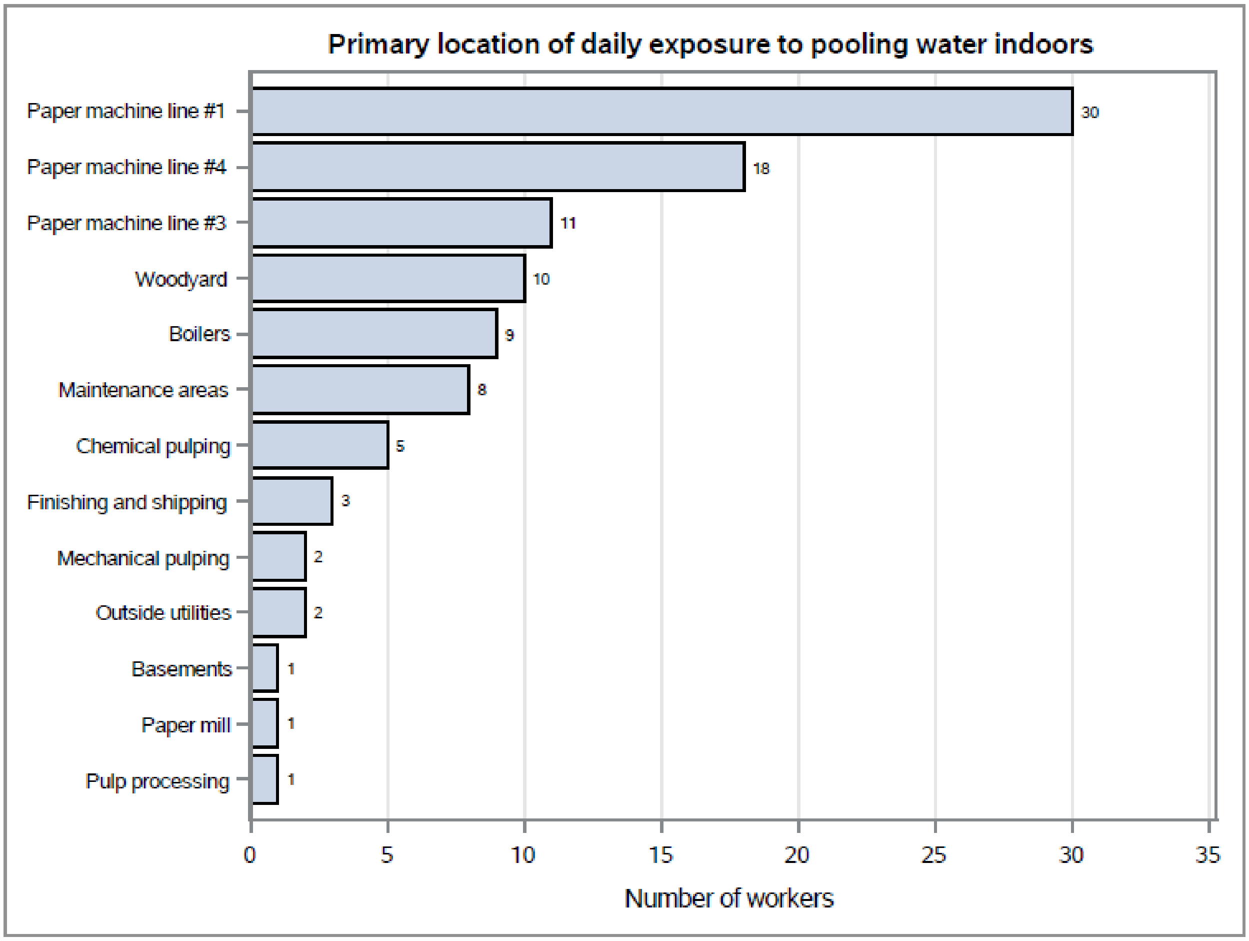

Supplemental Figure 4. Primary location of daily exposure to pooling water indoors for mill workers.
