# Supplementary material for: Assessment of environmental risk factors for blastomycosis during a large outbreak at a Michigan paper mill
Source: PLoS One. 2025 Sep 23;20(9):e0332398. doi: 10.1371/journal.pone.0332398 (PMC12456783; doi:10.1371/journal.pone.0332398)
Supplement: S2 Table — (PDF) [file pone.0332398.s008.pdf]

Supplemental Table 2. Environmental samples and PCR/fungal culture results for *Blastomyces* and other fungi.

Sample Type	Number of Samples	% positive for general fungi with PCR (ITS3/4)	% positive for general fungal growth	% Positive for *Blastomyces* (BAD-1)
Indoor surface dust	344	62	95	0
Outdoor soil/bulk samples	122	100	93	0
HVAC filter samples	66	95	25	0
Water from HVAC system	1	100	0	0
