# Supplementary material for: Assessment of environmental risk factors for blastomycosis during a large outbreak at a Michigan paper mill
Source: PLoS One. 2025 Sep 23;20(9):e0332398. doi: 10.1371/journal.pone.0332398 (PMC12456783; doi:10.1371/journal.pone.0332398)
Supplement: Attachment — Submitted filename: Blastomycosis Michigan paper mill_17062025.docx [file pone.0332398.s009.docx]

**"Blastomycosis during a large outbreak at a Michigan paper mill, 2023: A descriptive epidemiologic report"**

This paper provides discussed the largest outbreak of blastomycosis in the United States, happening at a paper mill in Michigan. The paper is well-written, however comments below need to be addressed:

1. The information on the of environmental sampling needs further clarification, including the sampling strategy (locations, frequency, sample types) and the rationale for focusing on certain environmental zones such as wood chip areas, ventilation systems).
2. “The interviewer-administered questionnaire collected self-reported data on work history and locations, environmental conditions, medical history, health symptoms, recent respiratory illnesses, blastomycosis-related information, and non-work-related activities…” – this sentence is confusing, is interviewer-administered or self-reported data collected by the authors?
3. The authors do not report whether serologic or skin tests (if available) were used to identify asymptomatic infection. There may be under reporting of mild or asymptomatic cases.
4. The authors do not report on the occupational hygiene assessments such as ventilation conditions and the use of PPE.
5. Are there any recent construction or maintenance activities during the outbreak?
6. The discussion does not sufficiently describe on how the findings are compared to prior blastomycosis outbreaks in the workplace settings.
